# 扩散加权成像在评估肺癌化疗效果中的应用价值

**DOI:** 10.3779/j.issn.1009-3419.2011.03.20

**Published:** 2011-03-20

**Authors:** 荣超 周, 铁链 于, 长超 冯, 力 马, 燕 王, 伟栋 李, 颖 王

**Affiliations:** 1 300052 天津，天津医科大学总医院放射科 Department of Radiology, General Hospital of Tianjin Medical University, Tianjin 300052, China; 2 300052 天津，天津医科大学总医院肿瘤科 Department of Radiology, the First Hospital of Qinhuangdao City, Qinhuangdao 060002, China; 3 060002 秦皇岛，秦皇岛市第一医院放射科 Department of Medical Oncology, General Hospital of Tianjin Medical University, Tianjin 300052, China

**Keywords:** 肺肿瘤, 磁共振扩散加权成像, 表观扩散系数, 化疗, Lung neoplasms, Diffusion-weighted imaging, Apparent diffusion coefficient, Chemotherapy

## Abstract

**背景与目的:**

磁共振扩散加权成像（diffusion-weighted imaging, DWI）能够在体检测水分子微观运动，对肿瘤治疗后微环境变化较为敏感。本研究旨在探讨DWI在监测、预测肺癌化疗效果中的应用价值。

**方法:**

对19例经病理确诊并接受化疗的肺癌患者，于化疗前一周和化疗开始后一个月分别行磁共振成像（magnetic resonance imaging, MRI）常规平扫及DWI检查。于横轴位T_2_加权像（T_2_ weighted imaging, T_2_WI）上测量肿瘤最大径（长径）、最大垂直径（短径）及长、短径平均值（平均径），并据此将肿瘤对化疗的反应分为完全反应（complete response, CR）、部分反应（partial response, PR）、稳定病变（stable disease, SD）和进展病变（progressive disease, PD）共四个疗效组。于DWI图象上测量化疗前和化疗后表观扩散系数（apparent diffusion coefficient, ADC）值，用*t*检验分析化疗前后ADC值及各疗效组化疗前ADC的差异。用*Pearson*相关系数分析化疗前后ADC值的变化率、化疗前ADC值与肿瘤各径线变化率之间的相关性。

**结果:**

与化疗前相比，肿瘤化疗后ADC值显著升高(1.482 ±0.456 *vs* 1.675±0.485, *P*=0.004)。化疗前后ADC变化率与其长径、短径及平均径的变化率呈正相关（*r*=0.635, *r*=0.612, *r*=0.539, *P* < 0.05）。PR组、SD组化疗前ADC值与其长径变化率呈负相关（*r*=-0.806, *r*=-0.632, *P* < 0.05）。

**结论:**

ADC值的变化能较敏感地反映肺癌化疗后的早期改变，结合形态学测量，有助于早期、动态监测化疗疗效。

## Abstract (en)

**Background and objective:**

DWI-MRI could detect the microscopic motion of water molecules which is sensitive to microcirculation change of many kinds of tumors after therapy. The aim of this study is to investigate the value of diffusion-weighted imaging (DWI) in monitoring and predicting lung cancer response to chemotherapy.

**Methods:**

Nineteen patients pathologically diagnosed as lung cancer were included in this study, all of whom received both conventional MRI and DWI examinations one week before and one month after the initiation of chemotherapy. The response of lung cancers to therapy were classified into four groups: complete response (CR), partial response (PR), stable disease (SD), and progressive disease (PD) based on the change of lesion diameter measured on axial T_2_-weighted MR imaging. Apparent diffusion coefficients (ADCs) were measured for each lesion at preand post-chemotherapy and were compared with t-test. The correlations between the percentage changes of ADCs and the percentage changes of diameters (long, short, and mean) were further evaluated with *Pearson* correlation coefficient, as well as that between pre-chemotherapy ADCs and the percentage changes of diameters.

**Results:**

The mean pre-chemotherapy ADCs was significantly higher than that of the post-chemotherapy ADCs (1.482 ± 0.456 *vs* 1.675±0.485, *P*=0.004). A positive correlation was found between the percentage changes of ADCs and the percentage changes of the long, short, and the mean diameter, respectively (*r*=0.635, *r*=0.612, *r*=0.539, *P* < 0.05). A negative correlation was found between pre-chemotherapy ADCs and the percentage change of the long diameter after chemotherapy in PR and SD group (*r*=-0.806, *r*=-0.632, *P* < 0.05).

**Conclusion:**

The change of ADCs can sensitively reflect the early change of lung cancer response to chemotherapy and has the potential ability to dynamically monitor the lung cancer response to chemotherapy combined with the morphological measurement.

联合化疗是中晚期不能手术肺癌患者的一线治疗方法^[1]^，准确可靠地评价化疗效果有助于临床及时有效地调整治疗方案，提高疗效。目前，磁共振扩散加权成像（diffusion-weighted imaging, DWI）已应用到多种类型肿瘤影像学检查中，包括鉴别良恶性病变、监测放疗或化疗的反应、鉴别治疗后改变与残存的活动性肿瘤、检测肿瘤复发^[2]^。目前据我们所知，利用DWI评价肺癌化疗疗效的研究报告在国内外尚未见正式报道。本研究目的是应用DWI评估肺癌患者化疗前后肿瘤最大径变化率和表观扩散系数（apparent diffusion coefficient, ADC）值的变化趋势，对DWI监测肺癌化疗疗效的可行性和临床价值进行初步探讨。

## 1 资料与方法

### 1.1 临床资料

收集2009年5月-2010年12月在天津医科大学总医院心胸外科及肿瘤科住院并接受化疗的中晚期肺癌患者19例，年龄45岁-74岁，平均（59.6±7.7）岁，男12例，女7例。其中鳞癌7例，腺癌7例，小细胞癌3例，不能进一步确定组织学分型的非小细胞癌2例。化疗前肿块最大径线范围2.22 cm-10.83 cm，平均（5.57±2.64）cm。经临床评估所有患者均不能行手术治疗。16例非小细胞肺癌分期：Ⅲa期6例，Ⅲb期10例。全部病例均于化疗前经病理证实，然后接受吉西他滨、长春瑞滨或依托泊苷+铂类药物化疗。

### 1.2 MRI检查方法及成像参数

对19例患者分别于化疗前一周和化疗开始后一个月行MRI常规平扫及DWI检查。使用GE HD-X 3.0T超导型磁共振扫描仪、Torsopa相控阵表面线圈。检查序列包括：①常规MRI平扫：快速回波快速自旋回波脉冲序列（fast relaxation fast spin echo, FRFSE）冠状位及轴位T_2_WI，采用呼吸触发技术，于轴位T_2_WI施加预饱和脂肪抑制技术得到轴位压脂T_2_WI；② DWI：扫描前先行ASSET校准序列扫描，然后采用单次激发自旋回波-回波平面成像序列（spin echo-echo planar imaging, SE-EPI）行轴位DWI扫描，b值取0 s/mm^2^、500 s/ mm^2^，同时在X、Y、Z轴三个方向上施加敏感梯度脉冲。

### 1.3 图像后处理及数据测量

使用AW4.3工作站的Functool 4.5.5软件包对图像进行后处理，获得肿瘤的DWI图、ADC图。参考T_2_WI和DWI图，在ADC图上选取病变中信号较均匀处放置兴趣区（region of interest, ROI），并注意ROI尽可能避开病灶边缘，面积约100 mm^2^-200mm^2^，测量ADC值，多次测量取平均值，分别获得化疗前后肿瘤ADC值^[3]^。

化疗前后肿瘤ADC值变化率=（化疗后ADC值-化疗前ADC值）/化疗前ADC值×100%。

### 1.4 疗效分组

选取轴位T_2_WI上可显示肿瘤最大截面的层面，测量化疗前后肿瘤最大径（长径）、最大垂直径（短径）及两者的平均值（平均径）。分别计算化疗前后肿瘤长径、短径及其平均径的变化率^[4]^。

肿瘤各径线变化率=（化疗前径线-化疗后径线）/化疗前相应径线×100%。

以横轴位目标病灶最大长径作为疗效评价的基线^[4]^，依据实体瘤治疗疗效评价标准^[5]^（Response Evaluation Criteria in Solid Tumors, RECIST）将其分为四组：①完全反应（complete response, CR）：肿瘤完全消失；②部分反应（partial response, PR）：肿瘤最大径减小至少30%；③稳定病变（stable disease, SD）：肿瘤最大径变化介于PR与PD之间；④进展病变（progressive disease, PD）：肿瘤最大径增长至少20%。

### 1.5 统计分析方法

使用SPSS 16.0统计分析软件。采用配对*t*检验分析化疗前后ADC值的变化是否具有统计学意义。采用两个独立样本*t*检验分析PR组、SD组化疗前肿瘤的ADC值的差异。采用两变量间相关分析计算*Pearson*相关系数，分析化疗前后肿瘤的长径、短径及平均径的变化率与ADC值变化率之间的相关性。以*P* < 0.05为有统计学差异。

## 2 结果

### 2.1 化疗前后肺癌DWI图和ADC图及其变化

化疗前19例肺癌在T_2_WI上肿块均呈稍高信号，DWI图上呈明显高信号，ADC图以蓝绿色区域为主。化疗后一个月，19例肺癌中有1例在常规MRI及DWI图上肿瘤均不再显示，符合CR组表现；12例肿瘤有不同程度缩小，其中符合PR组标准者8例，符合SD组标准者4例；5例肿瘤有增大，1例变化不明显，均符合SD组标准。本组肺癌化疗后无符合PD组标准者。化疗后PR组、SD组肿瘤于DWI图上仍呈高信号。9例有缩小的肿瘤在ADC图表现为以绿色或浅绿色区域为主，其余肿瘤在ADC图变化不明显（图 1）。

**1:**
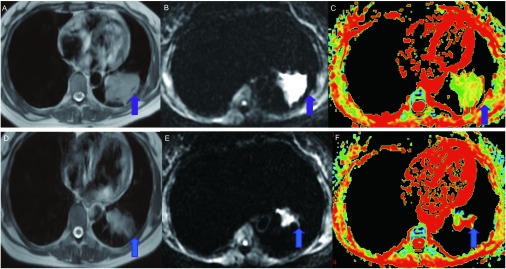
患者，女性，49岁，鳞癌。横轴位磁共振图，（化疗前）横轴位T_2_WI（A）、相应层面DWI图（B）及ADC图（C）。T_2_WI图左下叶不规则形长T_2_信号肿块，边缘不光整，DWI图上呈明显高信号（与邻近骨骼肌相比），ADC图以黄绿色区域为主，（箭头所示）；D-F（化疗1个月后）横轴位T_2_WI（D）、相应层面DWI图（E）及ADC图（F）。T_2_WI图肿块体积较前缩小，DWI图上呈高信号，ADC图以黄绿色伴红色区域为主，其中心部黄绿色区域范围较前减小（箭头所示）。DWI：磁共振扩散加权成像；ADC：表观扩散系数。 Transverse MRI images in a 49-year-old woman with squamous cell carcinoma of the lung (A, B, C: before chemotherapy; D, E, F: 1 month after chemotherapy). (A) T_2_-weighted MRI image showed an irregular hyperintense tumor (arrow); (B) On DWI image, the tumor was hyperintense in relation to adjacent skeletal muscle (arrow); (C) On ADC map, the tumor was depicted as an area of green and yellow(arrow); (D) T_2_-weighted MRI image showed that the size of tumor decreased (arrow); (E) On DWI images, the tumor was still hyperintense in relation to adjacent skeletal muscle (arrow); (F) On ADC map, the tumor was depicted an irregular contour with an area of green and yellow surrounding red. The area of color decreased (arrow). DWI: diffusion-weighted imaging; ADC: apparent diffusion coefficient.

### 2.2 肿瘤化疗前与化疗后ADC值比较CR组1例于化疗后

肿瘤不可见，故在统计分析中将其剔除。仅对其余18例肿瘤化疗前与化疗后ADC值及各径线进行比较（表 1）。化疗前后肿瘤ADC值差异具有统计学意义[(1.482 ±0.456) ×10^-3^ mm^2^/s *vs* (1.675±0.485)×10^-3^ mm^2^/s, *P*=0.004]，化疗后ADC值增高；化疗后肿瘤各径线较化疗前减小，但差异无统计学意义。

**1:** 18例肺癌化疗前与化疗后一个月ADC值及各径线比较 Comparison of ADCs and different diameter before and after chemotherapy

	Pre-therapy	Post-therapy	Difference value	*t*	*P*
ADC (10^-3^mm^2^/s)	1.482±0.456	1.675±0.485	0.193±0.247	3.324	0.004
Long diameter (cm)	5.683±2.663	4.746±2.584	0.937±0.875	1.071	0.292
Short diameter (cm)	4.488±2.344	3.680±2.075	0.808±0.738	1.096	0.281
Mean diameter (cm)	5.086±2.471	4.213±2.303	0.873±0.796	1.096	0.281

### 2.3 肿瘤化疗前后各径线变化率与ADC值变化率、化疗前ADC值的相关性

两变量间相关分析结果显示：肺癌化疗前后ADC值变化率与肿瘤长径、短径及平均径的变化率均呈正相关，而且ADC值变化率与肿瘤长径变化率之间的相关性相对最高（表 2）；PR组化疗前ADC值与化疗前后肿瘤长径变化率呈负相关（*r*=-0.806, *P*=0.016），SD组化疗前ADC值与肿瘤长径变化率呈负相关（*r*=-0.632, *P*=0.049, 7），PR组、SD组化疗前ADC值与肿瘤短径变化率、平均径变化率无明显相关性（表 3）。

**2:** 18例肺癌化疗前后ADC值变化率与各径线变化率的相关性分析 The correlation between the percentage change of ADCs and the percentage change of three sorts of diameters in 18 lung cancers underwent chemotherapy

Percentage change of ADCs (10^-3^mm^2^/s)	Percentage change of diameter (%)		*r*	*P*
14.49±16.49	Long diameter	17.04±22.45	0.635	0.005
	Short diameter	12.42±15.40	0.612	0.007
	Mean diameter	19.49±22.82	0.539	0.021

**3:** 不同疗效组肿瘤化疗前ADC值与各径线变化率的相关性分析 The correlation between pre-chemotherapy ADC value and the percentage change of three sorts of diameters in patient group with different response to therapy

Pre-therapy ADCs (10^-3^mm^2^/s)		Percentage change of diameter(%)		*r*	*P*
Partial response	1.359±0.296	Long diameter	33.88±19.66	-0.806	0.016
		Short diameter	22.38±17.16	-0.380	0.353
		Mean diameter	37.75±20.74	-0.677	0.065
Stable disease	1.580±0.549	Long diameter	3.35±13.59	-0.632	0.0497
		Short diameter	4.36±7.63	-0.439	0.204
		Mean diameter	4.82±10.58	-0.550	0.099

PR组化疗前ADC值低于SD组（表 3），但差异无统计学意义（*P*=0.095）。由于CR组仅1例，无法进行统计分析。

## 3 讨论

随着化疗方案和药物类型的不断更新，以铂类化疗药物为基础的联合化疗现被列为非手术肺癌患者一线治疗方案，能改善患者的生活质量，延长生存期。而放疗主要作为辅助治疗手段，起姑息治疗的作用，以改善患者生活质量。个体化治疗是近年新兴的一种治疗方法，具有较大潜力^[1, 6]^。因此如何监测肺癌的放化疗效果，以制定适合的个体化治疗方案至关重要。影像学方法是评估肿瘤治疗效果的重要手段之一。随着影像技术的快速发展，DWI在评价肿瘤治疗效果中的作用已引起广泛关注。DWI对肿瘤治疗后微环境变化较为敏感，可监测肿瘤发展过程中组织成分的变化及各种治疗措施引起的病理学改变^[2, 7]^，而且DWI检查无创，不必担心辐射问题，短期内可重复进行，具有独特优势。

DWI是目前唯一能够在体检测水分子微观运动的功能成像技术。生物组织内水分子的扩散运动与组织空间结构有关，分子粘滞度、核浆比、胞浆内大分子物质如蛋白质的分布、细胞内外结构之间膜通透性、主动转运等均可对其产生影响^[2, 8]^。对于实性肿瘤，肿瘤细胞密度对水分子扩散的影响更为重要，而细胞密度也是预测肿瘤预后的一个指标^[9]^。在细胞致密区域由于细胞密度增加，细胞外间隙减小和组织间液压力的升高以及细胞内结构的改变，导致水分子扩散运动明显受限，造成DWI信号升高，ADC值减低。肿瘤组织常较其起源组织细胞更加致密，因此DWI上表现为相对高信号^[9]^。有效的抗肿瘤治疗使肿瘤细胞坏死，细胞密度减小，细胞膜完整性消失，细胞外间隙增加，血管损伤，因而水分子扩散运动增加，DWI信号降低^[7, 10]^。许多DWI研究^[11]^发现，接受各种治疗后肿瘤组织的ADC值升高，并与其后的肿瘤消退或生长速度减慢高度相关，因此DWI具有可以早期评估肿瘤对治疗的敏感性的潜力。

DWI用于临床评价肿瘤治疗效果最初主要局限于脑肿瘤，随着EPI技术的出现，有关腹部肿瘤^[8, 12]^、软组织肿瘤^[13]^及骨肿瘤^[14]^等的DWI研究均已有文献报道。最近，刘颍等^[3]^研究显示出DWI定量指标ADC值可预测宫颈癌对放化疗的反应，化疗前肿瘤ADC值与放化疗后肿瘤长径变化率显著负相关。本文对19例不能手术而行联合化疗的中晚期肺癌患者进行了化疗前后DWI对照研究。通过对本组肺癌化疗前后ADC值及肿瘤各径线进行测量分析，结果显示肿瘤化疗后ADC值较化疗前明显升高（*P*=0.004），肿瘤各径线较化疗前减小，但差异无统计学意义（表 1），证实本组肿瘤ADC值的变化比形态学（长径、短径和平均径）变化更敏感地反映了肿瘤化疗后的早期改变，推测其原因与化疗后肿瘤ADC值的变化早于其形态学改变有关。本组肺癌ADC值的变化率与肿瘤长径、短径及两者平均径变化率均呈显著正相关，而且与长径变化率之间的相关性相对最高（表 2），表明肿瘤的长径是评估化疗疗效的一个有用的形态学测量指标。以上结果证实，ADC值能够较敏感地反映肺癌在化疗后组织内部结构的早期变化，在化疗过程中观察ADC值变化趋势和变化幅度并结合形态学的长径变化，将有助于动态监测疗效。

本研究也对肺癌化疗前的ADC值是否对化疗疗效具有预示意义进行了初步探讨。结果显示，PR组和SD组化疗前ADC值均与化疗前后肿瘤长径变化率呈负相关（表 3），表明化疗前ADC值的高低确实可能对化疗后肿瘤体积变化具有预示意义。但从表 3中可以看出，这种负相关在SD组仅勉强具有显著性意义（*P*=0.049, 7）。笔者在研究中观察到部分化疗前ADC值较低的肺癌患者，其化疗效果并不理想，ADC值的升高并不显著，究其原因可能与化疗后肿瘤细胞发生凝固性坏死并形成低灌注区，使其内肿瘤细胞处于缺氧和酸性环境中，影响化疗效果有关^[7]^。另一方面，不同组织学类型的肿瘤之间存在差异也可能对这种相关性产生影响，这可能也是本研究结果与其他作者的研究结果^[3]^不一致的原因之一。因此，对个体进行肺癌疗效预测时，仅凭借ADC值这一指标可能会产生一些偏倚。关于肺癌化疗前肿瘤ADC值对化疗疗效的预示价值有待扩大样本后进一步证实。

本研究的不足之处首先在于研究的病例数相对较少，无法进一步评价DWI在不同病理分型的肺癌化疗效果评估中的意义。其次，在化疗后仅进行了一次DWI扫描（第30天），因此未能评价肿瘤形态学改变与ADC值变化之间的确切动态关系。

综上所述，本研究初步证实肺癌化疗过程中DWI检查有助于早期监测治疗效果，使临床医师能快速、无创地监测治疗反应，及时发现对化疗不敏感的患者，以制定更有效的治疗措施。
